# Distinguishable Immunologic Characteristics of COVID-19 Patients with Comorbid Type 2 Diabetes Compared with Nondiabetic Individuals

**DOI:** 10.1155/2020/6914878

**Published:** 2020-09-29

**Authors:** Ruxing Zhao, Yujing Sun, Yongyuan Zhang, Weili Wang, Shouyu Wang, Chuang Wang, Jinbo Liu, Ling Gao, Zhao Hu, Jianchun Fei, Xinguo Hou, Huizhen Zheng, Li Chen

**Affiliations:** ^1^Department of Endocrinology, Qilu Hospital, Cheeloo College of Medicine, Shandong University, Jinan 250012, China; ^2^Institute of Endocrine and Metabolic Diseases of Shandong University, Jinan 250012, China; ^3^Jinan Clinical Research Center for Endocrine and Metabolic Diseases, Jinan 250012, China; ^4^Division of Medical Affairs, Qilu Hospital of Shandong University, Jinan 250012, China; ^5^Department of Endocrinology, Renmin Hospital of Wuhan University, Wuhan 430060, China; ^6^Department of Nephrology, Qilu Hospital, Cheeloo College of Medicine, Shandong University, Jinan 250012, China; ^7^Department of Anesthesiology, Qilu Hospital, Cheeloo College of Medicine, Shandong University, Jinan 250012, China

## Abstract

**Background:**

COVID-19 caused by severe acute respiratory syndrome coronavirus 2 (SARS-CoV-2) has threatened every civilian as a global pandemic. The immune system poses the critical interactive chain between the human body and the virus. Here, we make efforts to examine whether comorbidity with type 2 diabetes (T2D) affects the immunological response in COVID-19 patients.

**Methods:**

We conducted a retrospective pilot study investigating immunological characteristics of confirmed cases of COVID-19 with or without comorbid T2D. Two subcohorts of sex- and age-matched participants were eligible for data analysis, of which 33 participants were with T2D and the remaining 37 were nondiabetic (NDM). Cellular immunity was assessed by flow cytometric determination of surface markers including CD3, CD4, CD8, CD19, CD16, and CD56 in peripheral blood. Levels of C reactive protein, immunoglobulin (IgG, IgM, IgA, and IgE), and complements (C3, C4) were detected by rate nephelometry immunoassay. And Th1/Th2 cytokines (IL-2, IL-4, IL-6, IL-10, TNF-*α*, and IFN-*γ*) were detected by Cytometric Bead Array.

**Results:**

Neutrophil counts were found to be significantly higher in the T2D group than in the NDM group and had a significant relevance with clinical severity. Lymphocyte frequencies showed no significant differences in the two groups. However, the proportions and absolute counts of T, Tc, Th, and NK cells decreased in both groups to different degrees. An abnormal increase in neutrophil count and a decrease in lymphocyte subpopulations may represent risk factors of COVID-19 severity. The level of IgG, IgM, IgA, C3, and C4 showed no significant difference between the two groups, while the IgE levels were higher in the T2D group than in the NDM group (*p* < 0.05). Th1 cytokines including IFN-*γ*, TNF-*α*, and IL-6, as well as CRP, appeared significantly higher in the T2D group.

**Conclusions:**

The COVID-19 patients comorbid with T2D demonstrated distinguishable immunological parameters, which represented clinical relevancies with the predisposed disease severity in T2D.

## 1. Introduction

From January, 2020, we have been facing an unprecedented outbreak of coronavirus infectious disease-19 (COVID-19), which is now threatening every civilian in the world [1]. COVID-19 is caused by a novel severe acute respiratory syndrome coronavirus 2 (SARS-CoV-2) [2, 3]. Although COVID-19 leads to mild flu-like symptoms in the majority of affected patients, the disease may cause severe or even frequently lethal complications such as acute respiratory distress syndrome (ARDS) and multiorgan dysfunction (MODS) [1, 2, 4, 5]. And the coronavirus, including SARS-CoV-2, may likely pose a continuous threat to human health in the future [6]. Considering the infectivity of the virus, we must be prepared for further challenges. More knowledge on the pathogen and the host immune response is needed to develop more effective public health measures. Multimorbidity is also among the most important consideration for future control of the pandemic.

In parallel, T2D is one of the largest noncommunicable disease epidemics worldwide, whose increase is also in an uncontrolled and explosive manner [7, 8]. T2D is well established with alterations in both adaptive and innate immune systems, thus increasing the risk of susceptibility to most kinds of infections [9, 10]. Up to now, T2D is one of the most important comorbidities linked to the severity of all three known human pathogenic coronavirus infections, including SARS-CoV-2 [2, 10–12]. Previous data suggested that diabetes might likely be one of the most important comorbidities linked with COVID-19 [13]. It is known that 20–50% of patients in the current coronavirus COVID-19 pandemic had diabetes [14, 15]. Patients with T2D are supposed to have an increased risk of more severe outcome during infection [15]. Thus, an understanding of the intricate pathways responsible for the pathogenesis and complications in T2D and the development of strategies to enhance and stabilize the immune system are both urgently needed to prevent coinfections and comorbidities in COVID-19 patients with T2D [11, 15]. There are now more and more studies comprehensively demonstrating the clinical and laboratory parameters in COVID-19 with and without diabetes [16–19]. However, data on specific immunological changes in COVID-19 patients comorbid with T2D are yet limited [12].

In this retrospective pilot study, we examined the population of leukocytes and lymphocyte subsets, humoral immunity, infection-related biomarkers, and inflammatory cytokines in two subcohorts of sex- and age-matched clinically and laboratory-confirmed cases of COVID-19. We made efforts to check whether comorbidity with T2D affects the immunological parameters during diagnosis and management of COVID-19.

## 2. Materials and Methods

### 2.1. Participants and Ethic Statement

This study was a retrospective cohort study of hospitalized patients admitted to the People's Hospital of Wuhan University, one of the major hospitals nationally designated to provide medical care for COVID-19 patients in Wuhan, from February 5, 2020, to March 10, 2020. All participants enrolled were confirmed cases of COVID-19 diagnosed in compliance with the Guidelines for Diagnosis and Management of COVID-19 (6th edition) issued by the National Health Committee of China. Respiratory specimens were collected and then shipped to designated authoritative laboratories to detect the SARS-CoV-2 as previously reported [1, 5]. The positive finding of viral nucleic acid was considered essential for the enrollment. A detailed clinical record was registered for each subject, including history of COVID-19 disease, positive physical examination findings, and laboratory examinations. And a total of 70 participants were eligible and consecutively enrolled for the study, including two subcohorts of sex- and age-matched patients with and without T2D (referred as the T2D and NDM groups, *n* = 37 and *n* = 33, respectively) in search of immunological differences.

Patients with uncertain diagnosis of either type 2 diabetes (confirmed or suspected type 1 diabetes or other special types of diabetes) or COVID-19 or other unknown situations were excluded from the study. Exclusion criteria also included any clues of autoimmune disorders or tumors. All participants did not receive immunosuppressive or immunomodulatory drugs for at least 3 months before blood sampling.

This experiment was approved by the ethics committee of Qilu Hospital of Shandong University (No. 2020010). The data are kept anonymous; therefore, the requirement of written informed consent was waived. The study was conducted in compliance with the Declaration of Helsinki principles for ethical research.

### 2.2. Clinical Variables

Detailed clinical data were collected, including age, sex, exposure history, comorbid conditions, symptoms, and laboratory results. Clinical data including classification of disease severity and outcome were obtained from electronic medical records. Disease severity was categorized into mild, moderate, severe, and critically ill cases according to the above Guidelines for Diagnosis and Management of COVID-19. Due to the low incidence rate of critically ill cases in the cohort, we combined severe and critically ill cases as one classification—severe. Specimens, including sputum, blood, and urine were cultured to identify pathogenic bacteria or fungi secondary to or mixed with the SARS-CoV-2 infection. Mixed bacterial infection was diagnosed depending on both clinical manifestations and positive laboratory findings of microbiological examination and/or significantly increased procalcitonin (PCT > 0.1 ng/mL). Well-trained attending physicians were responsible for the diagnostic procedures, interpretation of laboratory analyses, and clinical decision during the patients' in-hospital stay. The data collection forms were reviewed independently by two researchers and analyzed in a blinded manner.

### 2.3. Laboratory Evaluation

Cellular immunity was assessed by multicolor flow cytometric determination of surface markers using human monoclonal anti-CD3-fluorescein isothiocyanate (FITC), anti-CD4-phycoerythrin (PE), anti-CD8-allophycocyanin (APC), anti-CD19-PE, anti-CD16-APC, and anti-CD56-PE antibodies (BD Multitest), for determination of proportions and numbers of total T (CD3+), helper T (Th, CD3+CD4+), cytotoxic T (Tc, CD3+CD8+), Natural Killer (NK, CD3-CD16+CD56+), and B (CD3-CD19+) cell subsets. All samples were examined by a BD FACSCanto II Flow Cytometer. Data were analyzed by FlowJo v10.0.

Serum levels of C reactive protein (hypersensitive, hsCRP), immunoglobulin (IgG, IgM, IgA, and IgE), and complements (C3, C4) were detected by rate nephelometry immunoassay (N Antiserum to Human Ig Kit series, Siemens, Germany). The plasma levels of cytokines (IL-2, IL-4, IL-6, IL-10, TNF-*α*, and IFN-*γ*) were detected by Cytometric Bead Array using the human Th1/2 cytokine kit II (BD Ltd., USA). All tests were conducted according to the manufacturer's instructions.

### 2.4. Statistical Analysis

Briefly, continuous parameters were presented as the mean ± SD or median according to data distribution. The classification variable was presented as a count (%). The statistical difference between two groups was determined by nonpaired Student's *t*-test unless the data were not normally distributed, in which case Mann-Whitney's *U* test was used instead. The chi-squared goodness-of-fit (Fisher's exact) test was used for the comparison of incident rates and proportions for categorical variables. The odds ratio was calculated in a 2 × 2 arranged table with Fisher's exact test. Spearman's correlation analysis was conducted between pairs of parameters to evaluate preliminarily the possible correlation. Ordinal multinomial logistic regression analysis was used to determine the associations of clinical and laboratory parameters with disease severity (mild, moderate, and severe/critical ill). For binary regression analysis, mild and moderate cases were defined as nonsevere and severe and critically ill cases were defined as severe. SPSS18.0 or GraphPad Prism 5.0 was used to perform all tests and generate values. A *p* value of less than 0.05 was considered statistically significant.

## 3. Results

### 3.1. Baseline Demographic Characteristics of COVID-19 Patients with and without T2D

Seventy patients met the inclusion criteria and were eligible for the study, of which 37 patients (52.9%) with diabetes belonged to the T2D group and 33 patients (47.1%) without diabetes belonged to the NDM group, respectively. There were no significant differences between the two groups among the demographic parameters, including age (average age or age distribution) and sex. Participants in both groups had underlying chronic medical conditions including a high prevalence of cardiovascular diseases (42.4% and 77.8%, respectively, including hypertension in this category). And we did find statistically significant differences concerning the comorbidity rates of these chronic diseases in our study cohort (shown in Table 1).

Previous literature had suggested that T2D might be associated with a higher risk of developing more severe types of infection [2, 11, 15]. However, there were no systemic data supporting the fact. In this retrospective cohort study, we also observed a significantly lesser proportion of mild types (18.9% vs. 48.5%) and more cases of severe types (24.3% vs. 12.1%) of infection in the T2D group compared with the NDM group (*p* = 0.038). In ordinal multinomial logistic regression analysis, comorbidity with T2D was for the first time proven to be a significant risk factor for having more severe types of infection (OR: 3.498 (1.369-8.938); *p* = 0.009^∗^). And the association was still statistically significant after adjusting for age and sex (OR: 3.388 (1.320-8.698); *p* = 0.011^∗^). Besides, patients from the T2D group also had a significantly higher risk of mixed bacterial infection than patients from the NDM group (see Table 1).

### 3.2. Distinguished Hematological Characteristics of COVID-19 in the T2D Group and Its Clinical Relevance

Here, we compare the hematological parameters between the two subcohorts. Patients with COVID-19 often had anemia, leukocytopenia, lymphocytopenia, and an increased monocyte count as previously reported [1, 2, 20]. Surprisingly, there were significant elevations in blood levels of leukocyte and neutrophil counts compared with NDM (shown in Table 2). Eosinophil count was decreased in T2D compared with NDM (0.08 vs. 0.02; *p* = 0.038^∗^). The T2D group also showed a significantly higher incidence of abnormal leukocytosis (16.22% vs. 0%; *p* = 0.0294^∗^) and an even more remarkably higher incidence of neutrophilia (OR: 11.85 (1.705 to 132.3); *p* = 0.0074^∗^). However, blood levels of lymphocyte count seemed lower in the T2D group than in the NDM group, but the current data failed to provide statistical significance (0.98 vs. 1.43; *p* = 0.097). There were no differences in monocyte (0.45 vs. 0.52; *p* = 0.2641) or basophil count (0.02 vs. 0.03; *p* = 0.960) between NDM and T2D patients. Since our data indicated that COVID-19 patients comorbid with T2D had a significantly higher incidence of mixed bacterial infection, we further analyzed whether the increased incidence of neutrophilia was associated with bacterial infection in a 2 × 2 arranged table with Fisher's exact test. The current data did not show correlation between neutrophilia and bacterial infection in either total participants or two subcohorts (*p* = 0.7601). The abnormal increase in neutrophil count might thus be associated with comorbidity with T2D and independent of bacterial infection.

**Figure 1:**
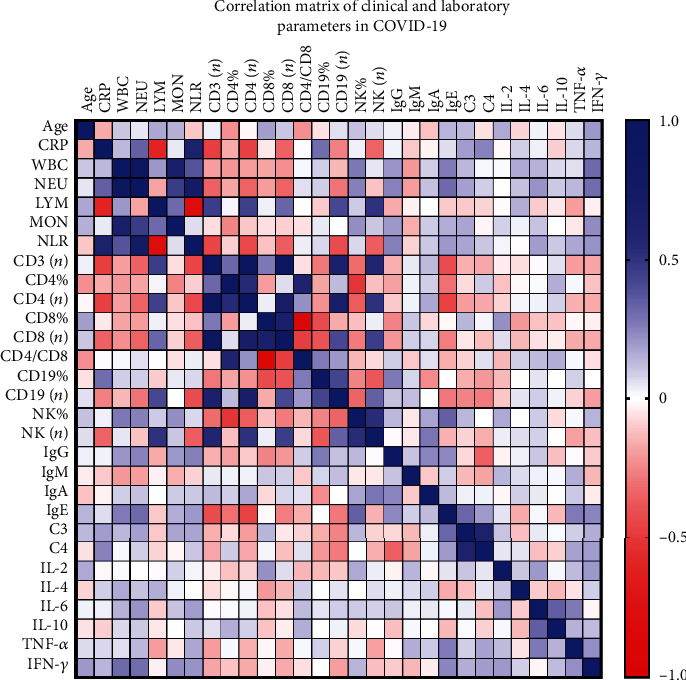
Correlation matrix of clinical and laboratory parameters in COVID-19 Spearman's correlation coefficients between two pairs of variables is shown in the heat map. The correlation coefficients are represented in terms of the change of the intensity of red/blue color, as shown in the color bar.

The results above indicate distinguishable changes in the white cell subpopulation of COVID-19 patients with T2D. Correlation analysis also revealed possible links between an abnormal increase in neutrophil count with decreased T (*r* = −0.348; *p* = 0.003^∗^), Th (*r* = −0.342; *p* = 0.004^∗^), Tc (*r* = −0.344; *p* = 0.004^∗^), and B (*r* = −0.288; *p* = 0.016^∗^) cell count and NK (*r* = −0.252; *p* = 0.035^∗^) cell proportion, as well as a positive link with IgG (*r* = 0.249; *p* = 0.043^∗^), IgE (*r* = 0.316; *p* = 0.024^∗^), and IFN-*γ* (*r* = 0.305; *p* = 0.010^∗^) (shown in Figure 1). As increases in neutrophil counts and neutrophil-to-lymphocyte ratio (NLR) were most recently established as predicative markers of severity during SARS-CoV-2 infection [20], we further conducted logistic regression analysis to verify whether neutrophilia represents an independent risk factor for adverse outcomes of COVID-19 in T2D. Neutrophil count was established as an independent risk factor associated with severe (and critically ill) types of COVID-19 in a binary univariate regression model (OR: 1.23 (1.029-1.527); *p* = 0.0314^∗^) after adjusting for age, sex, and mixed bacterial infection, which indicated that an increase of one thousand neutrophils per microliter of peripheral blood was associated with a 1.23-fold increase of risk of developing severe types (instead of mild or moderate types) of COVID-19. An increase in NLR did not represent a higher risk or significance in our study (OR: 1.095 (0.986-1.235); *p* = 0.1027). Interestingly, when comorbidity with T2D was introduced into the model, neutrophil count would lose its significant relevance. These preliminary data suggested that an abnormal increase in neutrophil count and its contribution to COVID-19 severity may be associated with dysregulated immune response in T2D.

### 3.3. Differences in Cellular Immunity in the T2D Group Compared with the NDM Group and Its Clinical Relevance

**Figure 2:**
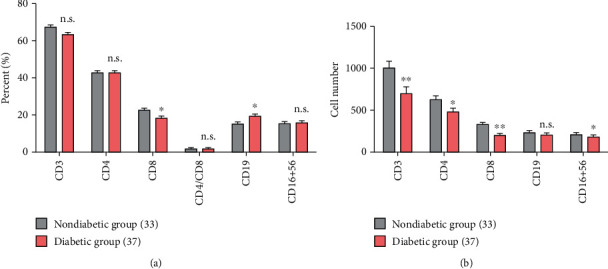
Comparisons of proportions and cell counts of lymphocyte subpopulations between COVID-19 patients with and without diabetes. Note: n.s.: *p* > 0.05; ^∗^*p* < 0.05; ^∗∗^*p* < 0.01.

As shown above, the total lymphocyte levels had no significant difference between the two groups. However, both groups had a high incidence of lymphopenia. Previous data had revealed that dysregulation of immune response, especially T lymphocytes, might be highly involved in the pathological process of COVID-19 [20]. We felt obliged to further compare and analyze the subsets of lymphocytes in two subcohorts. Sustained decrease in total T (CD3+) cells, Th (CD4+) cells, Tc (CD8+) cells, and NK (CD16+CD56+) cells was often observed in the current cohort of COVID-19 patients. The decrease of absolute amounts (cells per microliter of peripheral blood) of total T cells, Tc cells, Th cells, and NK cells was more significantly remarkable in the T2D group than in the NDM group (shown in Table 3 and Figure 2(a)). A decrease in Tc cells appeared to be more dominant; thus, the Th/Tc (CD4+/CD8+) ratio was often increased in COVID-19 patients. However, we did not observe a significant difference in the Th/Tc ratio between the two groups. Some of the patients had an increased number and/or proportion of B cells, which might represent a normal response to viral infection. Here, we found statistical difference in the proportion but not in the number of B cells between the T2D group and the NDM group (see Table 3 and Figure 2).

Similar tendencies were also observed in the incidences of parameter abnormity between the two groups. The degree and incidence of decreased Tc cells turned out to be a more distinguished dysregulated cellular component in COVID-19, which is consistent with previous studies [5, 21]. 36.4% of patients without diabetes and above half (66.7%) of T2D patients had a decreased Tc cell count (*p* = 0.040^∗^). Patients from the T2D group also had a significantly higher incidence of decreased Tc cell proportion than those from the NDM group (46.2% vs. 12.1%; *p* = 0.008^∗^). The T2D group also had a remarkably higher incidence of decreased NK cell count than the NDM group (35.89% vs. 6.06%; *p* = 0.001^∗^). However, when considering the frequencies of the cell subpopulation, there were less patients with decreased Th cell proportion in the T2D group than in the NDM group (2.56% vs. 18.2%; *p* = 0.044^∗^). Although the T2D group had a lower median level of Th cell count, they had a lower incidence of decreased Th cell count at the same time (see Table 3). Currently, using ordinal multinomial logistic regression models, univariate analysis revealed that only Th cell count might have potential negative relevance (OR: 0.176 (0.034-0.907); *p* = 0.0379^∗^) with the COVID-19 severity. When adjusted by age and sex, the relevance remained statistically significant and even more remarkable (OR: 0.137 (0.024-0.790); *p* = 0.0261^∗^). Age and sex might have an influence on the absolute number of Th cell count. When comorbidity with T2D was introduced into the model, T2D still represented a significant risk factor for more severe types of disease (OR: 2.760 (1.049-7.266); *p* = 0.0398^∗^), but again Th cell count lost its significance (*p* = 0.086). In the correlation analysis, Th cell count correlated with many other parameters involving antiviral response as well as inflammatory markers. It positively correlated with lymphocyte count, Tc cell count, B cell count, and NK cell count significantly, but it negatively correlated with CRP levels and neutrophil count (shown in Figure 1). As an important regulatory immune component, Th cells may play a double-edged role in antiviral response and uncontrolled inflammation. The abovementioned data supported the possibility that there were discrepant and even divergent cellular immune responses to SARS-CoV-2 infection in patients comorbid with T2D.

### 3.4. Differences in Serum Immunoglobulin and Complements in the T2D Group Compared with the NDM Group

Humoral immunity plays important roles in antiviral response. In this study, we observed changes in serum levels of immunoglobulin and complements in COVID-19. Patients had more often increased levels of IgG, IgE, and complement C4, but decreased levels of C3 (shown in Table 4). The average levels of the IgG, IgM, IgA, C3, and C4 showed no significant difference between the two groups. But abnormally higher IgE levels were found to be statistically significant in the T2D group compared with the NDM group. And there were also more patients with an increased level of IgG in the T2D group than in the NDM group. Moreover, serum IgE negatively correlated with Th and B cell counts significantly but positively correlated with neutrophil count (*p* < 0.05^∗^). In search of the clinical relevance of the immunoglobulin and complements with disease severity, we further conducted univariate logistic regression analysis involving IgG, IgM, IgA, IgE, C3, and C4, but none of them indicated statistical significance (data not shown).

### 3.5. Differences in Serum Th1/Th2 Cytokines of Patients in the T2D Group Compared with the NDM Group

As cytokine storms played a critical role in the deterioration of COVID-19, we also detected the Th1/Th2 cytokines including IL-2, IL-4, IL-6, IL-10, TNF-*α*, and IFN-*γ* in patients infected with COVID-19. As shown in Table 5, we did not find statistical difference in the levels of the Th2 cytokines including IL-2, IL-4, and IL-10. However, serum levels of IFN-*γ*, TNF-*α*, IL-6, and CRP were all significantly higher in the T2D group than in the NDM group (*p* = 0.005, *p* = 0.0001, *p* = 0.008, and *p* = 0.015, respectively). What is more, increased serum levels of CRP correlated significantly not only with increased neutrophil count (and NLR) but also with decreased lymphocyte, T cell, Th, Tc, B cell, and NK cell counts (*p* < 0.05^∗^, shown in Figure 1). However, none of them showed statistical significance considering the clinical relevance of the cytokines with COVID-19 severity by univariate logistic regression analysis.

## 4. Discussion

The ongoing pandemic of COVID-19 is now a global health-threatening crisis [2, 3]. Within the past half year, we have accumulated limited knowledge of the novel infectious disease. The immune response is believed to be most involved in the pathological process of COVID-19 [5, 22–24]. The effectual host immune response including innate and adaptive immunity against SARS-CoV-2 is crucial to control and resolve the viral infection [3, 22, 24]. However, the severity and outcome of COVID-19 might also be associated with dysregulated immune response and excessive production of proinflammatory cytokines [5, 23, 25, 26]. The immune system is impaired during the disease, characterized by leukocytopenia (esp. lymphocytopenia) and uncontrolled systemic inflammatory response in the severe cases [20, 23, 25]. Most recent data demonstrated that COVID-19 might affect lymphocytes, especially T lymphocytes [20, 21]. The absolute number of T lymphocytes, CD4+ T cells, and CD8+ T cells decreased in nearly all the patients and was markedly lower in severe cases [5, 20, 27]. And these potential immunological markers may be of importance due to their correlation with disease severity in COVID-19 [5, 23, 25, 26, 28].

T2D is well established as an inflammatory disease characterized by immune disturbance (e.g., long-term activation of the innate immune system) and systemic low-grade inflammation [29, 30]. The basal immune status in T2D is dysregulated, which is believed to affect normal immune response against viral infection. In this retrospective pilot study, we made efforts to find out whether comorbidity with T2D was related to different immunological parameters during diagnosis and management of COVID-19. Patients with COVID-19 often had leukocytopenia, lymphocytopenia, and an increased monocyte count as previously reported [1, 2, 20]. However, we first revealed an abnormal increase in leukocyte (esp. neutrophil) count in patients comorbid with T2D compared with NDM. The T2D group also showed a significantly higher incidence of abnormal neutrophilia, which was primarily proven to be independent of secondary or combined bacterial infection. These data implied a dysregulated neutrophil response to SARS-CoV-2 infection in T2D patients. Neutrophil count was further established as an independent risk factor associated with severe (and critically ill) types of COVID-19, adjusted by age, sex, and mixed bacterial infection. Increased neutrophil or neutrophil-to-lymphocyte ratio (NLR) is established as an independent risk factor and prognostic tool to predict the clinical outcomes of COVID-19 [22, 31–33]. Our data revealed that COVID-19 patients comorbid with T2D have distinguishable changes in the white cell subpopulation characterized by an intrinsically increased neutrophil count. And the abnormal increase in neutrophil count and its contribution to COVID-19 severity may be associated with innate dysregulation of immune response in T2D [33].

Previous studies had revealed that the dysregulation of cellular immune response, especially T lymphocytes, might be highly involved in the pathological process of COVID-19 [20, 25, 27]. Although the total lymphocyte count or the incidence of lymphopenia demonstrates no significant difference between the two groups, we observed distinguishable differences in the subpopulations of lymphocytes. The sustained decrease in total T cells as well as Th and Tc subsets and NK subsets were all more remarkable in the T2D group than in the nondiabetic group. Previous studies have demonstrated that the absolute number of T cell subsets was decreased in most patients, especially in severe cases [5, 20–22, 25, 27]. Similar but not perfectly consistent tendencies were observed in the incidences of parameter abnormity in this study. 36.4% of patients without diabetes and 66.7% of T2D patients had a decreased Tc cell count. Patients from the T2D group also had a significantly higher incidence of decreased Tc cell proportion and NK cell count compared to those from the NDM group. And we found a significantly higher proportion but not a higher number of B cells in T2D patients. Conflictingly, T2D patients had a lower median level of Th cell count, but less patients in the T2D group had decreased Th cell proportion compared to those in the NDM group. This fact was due to a discrepant distribution of Th levels in T2D patients. And it reminded us to pay attention not only to the absolute levels of the immunological parameters but also to the incidence of abnormity in those parameters. Therefore, we analyzed differences in not only absolute values but also in the abnormal rates of the laboratory parameters between two subcohorts of patients. There were discrepant and even divergent cellular immune responses to SARS-CoV-2 infection in patients comorbid with T2D. And univariate analysis laid more stress on the relevance of the Th subset with disease severity in the current data. Th cell correlated positively with lymphocyte count, Tc count, B cell count, and NK cell count significantly, but correlated negatively with CRP levels and neutrophil count. Series of studies have confirmed that there is a skewed proinflammatory T cell compartment, especially Th subsets in the peripheral blood of T2D patients [34–36]. Th cells may play a double-edged role in antiviral response and uncontrolled inflammation. Besides, T2D is also accompanied by a baseline decrease in NK cell function [34, 37]. And both cytotoxic lymphocytes including Tc and NK were more remarkably impaired in T2D compared with nondiabetic patients in our study. Since Tc and NK interact intensively with the virus and play dominant roles in the clearance of virally infected cells, NK cells are also major immune regulators which bring order and discipline to the infected tissue microenvironment [38]. Decreased NK cell count may also explain the uncontrolled expansion and activation of other immune effectors [20, 38]. Therefore, the dysregulated basal levels and discrepant response of lymphocyte subpopulations in T2D are postulated to have a critical role in the severity and adverse outcome of the disease [20, 39, 40].

As an important regulatory immune component, Th cells regulate antigen presentation and immunity against intracellular SARS-CoV-2 through IFN-*γ* production. Dysregulated T cell function relieves the inhibition on the innate immune system leading to secretion of high amounts of inflammatory cytokines in what is known as a “cytokine storm” [41]. We also investigated the Th1/Th2 cytokines in patients with and without diabetes. We did not observe statistical difference in the levels of the Th2 cytokines including IL-2, IL-4, and IL-10. However, serum levels of IFN-*γ*, TNF-*α*, and IL-6, as well as CRP, were all significantly higher in the T2D group than in the NDM group. And increased CRP also correlated significantly with increased neutrophil count and negatively with lymphocyte, T cell, Th, Tc, B cell, and NK cell counts in this study. Therefore, Th1 cells may induce the recruitment of neutrophils and macrophages by producing inflammatory cytokines [42], which in turn contribute to the hyperinflammatory response observed in patients with diabetes [39]. Monoclonal antibody therapy targeting Th1 cytokines might be helpful especially in patients with T2D.

Moreover, T2D patients also demonstrate distinguishable differences in humoral immunity. COVID-19 patients had more often increased levels of IgG, IgE, and complement C4, but a decreased level of C3 in our study. The average levels of IgG, IgM, IgA, C3, and C4 showed no significant difference between the two groups. But abnormally higher IgE levels were observed in the T2D group. Serum IgE negatively correlated with Th and B cell counts significantly, but it positively correlated with neutrophil count in our cohort. Recent advances suggested that an increase in type 2 (antihelminths) effectors including IgE and eosinophils also reflected the development of a maladapted immune response profile and was associated with severe COVID-19 outcome [43]. These changes may represent a dysregulated humoral immune response in T2D and might be associated with the proinflammatory status and adverse outcome during infection [43].

In conclusion, the COVID-19 patients comorbid with T2D demonstrated distinguishable immunological parameters during the infection. Some of the discrepancies demonstrated clinical relevance with the predisposed adverse outcome of COVID-19 in T2D. Dysregulated baseline status and discrepant response of both cellular and humoral immunity in T2D are postulated to have a critical role in the severity and adverse outcome of the disease. The current data could shed some light on the understanding of the unique response to SARS-CoV-2 infection in T2D and thus provide useful clues for the development of more effective strategies.

## Figures and Tables

**Table 1:** Basal characteristics of patients infected with COVID-19.

	NDM (*n* = 33)	T2D (*n* = 37)	*p* value
Age distribution			
25-49, *n* (%)	6 (19.6%)	5 (13.5%)	0.592
50-64, *n* (%)	10 (30.3%)	15 (40.5%)	0.372
≥65, *n* (%)	17 (51.5%)	17 (45.9%)	0.641
Age (years)	62.1 ± 16.2	63.4 ± 12.8	0.690
Sex			
Female, *n* (%)	15 (45.5%)	18 (48.6%)	0.699
Severity classification			0.038^∗^
Mild type, *n* (%)	16 (48.5%)	7 (18.9%)	—
Moderate type, *n* (%)	13 (39.4%)	20 (54.1)	—
Severe cases, *n* (%)	4 (12.1%)	9 (24.3%)	—
Critically ill type, *n* (%)	0 (0.0%)	1 (2.7%)	—
Comorbidities at admission			
Mixed bacterial infection, *n* (%)	4 (12.1%)	12 (32.3%)	0.043^∗^
Cardiovascular diseases, *n* (%)	14 (42.4%)	21 (77.8%)	0.231
Digestive system disease, *n* (%)	2 (6.1%)	3 (8.1%)	0.895
Respiratory system diseases, *n* (%)	2 (6.1%)	3 (8.1%)	0.895
Chronic kidney disease, *n* (%)	2 (6.1%)	5 (18.5%)	0.521
Chronic liver disease, *n* (%)	1 (3.0%)	1 (2.7%)	0.628
HIV infection, *n* (%)	0 (0.0%)	0 (0.0%)	1.000
Septic shock, *n* (%)	0 (0.0%)	0 (0.0%)	1.000

**Table 2:** Laboratory findings of white cell subpopulations in patients with diabetes or not infected with COVID-19.

Variables		NDM (*n* = 33)			T2D (*n* = 37)			*p* value
(×10^9^/L)	Normal range	Median	Increased no. (%)	Decreased no. (%)	Median	Increased no. (%)	Decreased no. (%)	
WBC	3.5-9.5	4.74	0 (0.0%)	5 (15.2%)	6.05	6 (16.2%)	2 (5.4%)	0.015^∗^
NEU	1.8-6.3	2.50	1 (3.0%)	2 (6.1%)	4. 10	10 (27.0%)	0 (0.0%)	<0.001^∗^
LYM	1.1-3.2	1.43	0 (0.0%)	17 (39.5%)	0.98	3 (8.1%)	18 (48.7%)	0.097
ESO	0.02-0.52	0.08	0 (0.0%)	8 (24.3%)	0.02	0 (0.0%)	16 (43.2%)	0.038^∗^
BAS	0-0.06	0.02	2 (6.1%)	0 (0.0%)	0.03	1 (2.7%)	0 (0.0%)	0.960
MON	0.1-0.6	0.45	7 (21.2%)	0 (0.0%)	0.52	11 (29.7%)	0 (0.0%)	0.264

^1^WBC: white blood cell count; NEU: neutrophil count; LYM: lymphocyte count; ESO: eosinophil count; BAS: basophil count; MON: monocyte count. ^2^Note that the *p* values were calculated by comparisons of absolute values (medians or means) in this table.

**Table 3:** Status of cellular immunity in patients with and without type 2 diabetes.

Variables	Normal range	NDM (*n* = 33)			T2D (*n* = 37)			*p* value
		Median	Increased no. (%)	Decreased no. (%)	Median	Increased no. (%)	Decreased no. (%)	
T (%)	56-86	67.0	0 (0.0%)	4 (12.1%)	62.9	0 (0.0%)	9 (23.1%)	0.059
T (*n*/*μ*L)	723-2737	983	0 (0.0%)	9 (27.3%)	679	0 (0.0%)	19 (48.7%)	0.004^∗^
Th (%)	33-58	41.1	1 (3.0%)	6 (18.2%)	42.0	0 (0.0%)	1 (2.6%)	0.926
Th (*n*/*μ*L)	404-1612	604	0 (0.0%)	8 (24.2%)	451	0 (0.0%)	6 (15.4%)	0.029^∗^
Tc (%)	13-39	24.0	2 (6.1%)	4 (12.1%)	18.7	0 (0.0%)	18 (46.2%)	0.017^∗^
Tc (*n*/*μ*L)	220-1129	328	0 (0.0%)	12 (36.4%)	203	0 (0.0%)	22 (66.7%)	<0.001^∗^
Th/Tc	0.9-2.0	2.12	15 (45.5%)	3 (9.1%)	2.54	22 (56.4%)	0 (0.0%)	0.221
B (%)	5-22	13.4	4 (12.1%)	0 (0.0%)	19.4	12 (30.8%)	0 (0.0%)	0.018^∗^
B (*n*/*μ*L)	80-616	187	1 (3.0%)	2 (6.1%)	170.3	1 (2.6%)	5 (15.2%)	0.384
NK (%)	5-26	17.3	2 (6.1%)	0 (0.0%)	16.4	3 (7.7%)	3 (7.7%)	0.810
NK (*n*/*μ*L)	84-724	242	0 (0.00%)	2 (6.1%)	186	1 (2.6%)	14 (35.9%)	0.045^∗^

**Table 4:** Serum levels of immunoglobulins and complements in patients with and without type 2 diabetes.

Variables	Normal range	NDM (*n* = 33)			T2D (*n* = 37)			*p* value
		Median	Increased no. (%)	Decreased no. (%)	Median	Increased no. (%)	Decreased no. (%)	
IgG (g/L)	7-16	23.83	2 (6.5%)	1 (3.2%)	25.2	10 (27.8%)	1 (2.8%)	0.547
IgM (g/L)	0.4-2.3	1.03	0 (0.0%)	1 (3.2%)	1.02	2 (5.6%)	0 (0.0%)	0.680
IgA (g/L)	0.7-4.0	2.87	2 (6.5%)	0 (0.0%)	3.13	8 (22.2%)	1 (2.8%)	0.457
IgE (IU/mL)	<100	88.5	5 (16.1%)	0 (0.0%)	163.4	13 (36.1%)	0 (0.0%)	0.002^∗^
C3 (g/L)	0.9-1.8	1.03	0 (0.0%)	3 (9.7%)	1.01	1 (2.8%)	7 (19.4%)	0.402
C4 (g/L)	0.1-0.4	0.23	4 (12.9%)	1 (3.2%)	0.25	3 (8.3%)	1 (2.8%)	0.839

**Table 5:** Serum levels of Th1/Th2 cytokines in patients with and without type 2 diabetes.

Variables (pg/mL)	Normal range	NDM (*n* = 33)		T2D (*n* = 37)		*p* value
		Mean or median	Increased no. (%)	Mean or median	Increased no. (%)	
IL-2	≤11.4	4.09 ± 2.02	0 (0.00%)	3.84 ± 1.24	0 (0.00%)	0.516
IL-4	≤12.9	3.64 ± 0.82	0 (0.00%)	3.67 ± 0.95	0 (0.00%)	0.888
IL-6	≤20	5.27	4 (12.1%)	12.10	9 (24.3%)	0.008^∗^
IL-10	≤5.9	4.50	8 (24.2%)	5.05	5.05 (29.7%)	0.187
TNF-*α*	≤5.5	3.83	6 (18.2%)	6.43	19 (51.4%)	<0.001^∗^
IFN-*γ*	≤18	3.59	2 (6.1%)	8.83	3 (8.2%)	0.005^∗^

## Data Availability

The authors will make the primary data available under the premise of privacy protection, restricted usage, and duplication. Access to additional primary data will be considered by the authors upon request (rusingstar@163.com).
